# Supplementary material for: Aesthetic Impact of Orthognathic Surgery vs. Orthodontic Camouflage in Class II Division 1 Patients with Convex Facial Profile: A Follow-Up Using Combined Frontal and Profile Views
Source: J Clin Med. 2025 Jun 16;14(12):4277. doi: 10.3390/jcm14124277 (PMC12194542; doi:10.3390/jcm14124277)
Supplement: Supplementary file 1 [file jcm-14-04277-s001.zip › jcm-3638554-supplementary.pdf]

Aesthetic Impact of Orthognathic Surgery vs. Orthodontic Camouflage in Class II Division 1 Patients with Convex Facial Profile: A Follow-Up Using Combined Frontal and Profile Views

Simos Psomiadis, Iosif Sifakakis, Ioannis Iatrou and Nikolaos Gkantidis

Supplementary Table S1. Overview of the patient sample characteristics. (Reprint from Psomiadis et al. 2023[17] ; https://doi.org/10.3390/jcm13010091)

Treatment type	N (Sex)	Age (yrs) Mean±SD		Treatment duration (yrs) Mean±SD	Facial contour angle (°) Mean±SD			Overjet (mm) Mean±SD	
		T0	T1		T0	T1	T1-T0	T0	T1
Camouflage	18 (8M, 10F)	22.7±8.3	25.1±8.1	2.5±0.8	20.2±3.3	19.0±3.5	-1.2±2.1	7.6± 2.2	3.9±1.6
Surgery	18 (8M, 10F)	23.9±7.4	27.1±7.1	3.1±1.3	22.3±6.6	16.2±6.3	-6.2±3.9	8.9±2.8	3.6±1.9
P-value*	-	0.643	0.442	0.078	0.169	0.189	<0.001*	0.137	0.629

M: males, F: females, yrs: years, SD: standard deviation, T0: pre-treatment, T1: post-treatment
* Mann–Whitney U test, Bonferroni adjusted level of significance: $p < 0.01$

Supplementary Table S2. Additional patient sample characteristics.

Treatment type	N (Sex)[1]	ANB (°) Mean±SD		Overbite (mm) Mean±SD		Wits (mm) Mean±SD		Facial angle (FH-NPog) (°) Mean±SD	
		T0	T1	T0	T1	T0	T1	T0	T1
Camouflage	18 (8M, 10F)	5.5±1.5	5.2±2.1	3.3±1.9	2.0±0.8	4.0±2.2	3.1±2.7	85.9± 3.0	86.0±4.4
Surgery	18 (8M, 10F)	7.2±2.0	4.0±2.7	4.2±2.3	1.5±1.6	6.1±2.1	0.9±2.6	84.1±3.5	86.8±4.3
P-value*	-	0.018	0.275	0.090	0.961	0.008	0.031	0.145	0.715

M: males, F: females, SD: standard deviation, T0: pre-treatment, T1: post-treatment
* Mann–Whitney U test, Bonferroni adjusted level of significance: $p < 0.006$
[1] For the cephalometric variables, the sample size at T1 was smaller (Camouflage group: N=14, Surgery group: N=13) due to unavailable radiographs.

Supplementary Table S3. Agreement among rater groups assessed through the intraclass correlation coefficient (ICC; two-way mixed model, absolute agreement, results regarding average measures; 95% confidence intervals reported in parentheses).

Treatment group	Face	Lower face	Upper lip	Lower lip	Chin
Entire sample	0.92 (0.86, 0.96)	0.93 (0.89, 0.96)	0.95 (0.91, 0.97)	0.93 (0.88, 0.96)	0.92 (0.86, 0.96)
Camouflage	0.88 (0.76, 0.95)	0.89 (0.78, 0.95)	0.89 (0.77, 0.95)	0.90 (0.79, 0.96)	0.89 (0.79, 0.96)
Surgery	0.87 (0.73, 0.94)	0.89 (0.76, 0.95)	0.95 (0.90, 0.98)	0.90 (0.80, 0.96)	0.88 (0.75, 0.95)

Supplementary Table S4. Results of the ANOVAS testing the effect of photographic setup, rater group, and treatment group on the assessed changes from pre- to post-treatment condition.

Source	Dependent Variable	df	F	Sig.
Treatment group	Face[a]	1	145.68	<0.001
	Lower Face[b]	1	155.21	<0.001
	Upper Lip[c]	1	101.07	<0.001
	Lower Lip[d]	1	107.71	<0.001
	Chin[e]	1	124.80	<0.001
Rater group	Face	3	2.38	0.070
	Lower Face	3	2.09	0.102
	Upper Lip	3	0.19	0.902
	Lower Lip	3	3.80	0.011
	Chin	3	2.47	0.063
Photographic setup	Face	1	0.71	0.401
	Lower Face	1	1.61	0.206
	Upper Lip	1	0.52	0.471
	Lower Lip	1	0.17	0.684
	Chin	1	1.04	0.308
Treatment group × Rater group	Face	3	0.62	0.602
	Lower Face	3	0.34	0.798
	Upper Lip	3	0.12	0.945
	Lower Lip	3	0.29	0.829
	Chin	3	1.15	0.328
Treatment group × Photographic setup	Face	1	0.10	0.756
	Lower Face	1	0.33	0.566
	Upper Lip	1	0.06	0.813
	Lower Lip	1	0.20	0.658
	Chin	1	0.06	0.813
Rater group × Photographic setup	Face	3	0.22	0.881
	Lower Face	3	0.28	0.838
	Upper Lip	3	0.58	0.629
	Lower Lip	3	0.03	0.994
	Chin	3	0.55	0.646
Treatment group × Rater group × Photographic setup	Face	3	0.03	0.992
	Lower Face	3	0.27	0.846
	Upper Lip	3	0.24	0.870
	Lower Lip	3	0.20	0.895
	Chin	3	0.21	0.890

[a]R Squared = 0.36 (Adjusted R Squared = 0.33), [b]R Squared = 0.38 (Adjusted R Squared = 0.3), [c]R Squared = 0.28 (Adjusted R Squared = 0.24), [d]R Squared = 0.31 (Adjusted R Squared = 0.27), [e]R Squared = 0.34 (Adjusted R Squared = 0.30). df: degrees of freedom. F: F-value. Sig.: Significance shown as p-values.

Supplementary File S1. STROBE Checklist v4 for Cohort Studies.

Item	No.	Recommendation	Correspondence to the Study
**Title and abstract**	1	Indicate the study's design with a commonly used term in the title or the abstract. Provide in the abstract an informative and balanced summary of what was done and what was found.	Yes – The design is retrospective cohort and comparative, clearly mentioned in the abstract.
**Introduction**			
Background/rationale	2	Explain the scientific background and rationale for the investigation being reported.	Yes – The introduction thoroughly explains the background, significance of facial aesthetics, and the rationale for comparing treatments.
Objectives	3	State specific objectives, including any prespecified hypotheses.	Yes – The study clearly states its objective to reassess aesthetic outcomes and provides a specific hypothesis.
**Methods**			
Study design	4	Present key elements of study design early in the paper.	Yes – The paper identifies it as a retrospective cohort study using previously collected clinical data.
Setting	5	Describe the setting, locations, and relevant dates, including periods of recruitment, exposure, follow-up, and data collection.	Yes – Study setting and period (postgraduate orthodontic clinic, dates of data approval and

			collection) are described.
Participants	6	Give the eligibility criteria, sources and methods of selection of participants. Describe methods of follow-up.	Yes – Eligibility criteria, sampling method, and group matching are clearly explained.
Variables	7	Clearly define all outcomes, exposures, predictors, potential confounders, and effect modifiers.	Yes – Treatment type is the exposure; facial appearance change is the outcome. Confounders like rater type are analyzed.
Data sources/measurement	8	For each variable of interest, give sources of data and details of methods of assessment (measurement).	Yes – Data sources (photos, questionnaires), and assessment methods (VAS, ICC) are well detailed.
Bias	9	Describe any efforts to address potential sources of bias.	Yes – Efforts include randomization of photo orientation, blinded raters, and use of standardized photo processing.
Study size	10	Explain how the study size was arrived at.	Yes – Sample size justification is based on prior literature and post-hoc power analysis.
Quantitative variables	11	Explain how quantitative variables were handled in the analyses. If applicable, describe groupings.	Yes – Quantitative VAS scores analyzed using MANOVA and ANOVA; results are stratified by treatment and rater groups.
Statistical methods	12	Describe all statistical methods, including those used to control for confounding.	Yes – Statistical methods including MANOVA, ICC, post-hoc tests, and corrections for

			multiple testing are described.
**Results**			
Participants	13	Report numbers of individuals at each stage of study—e.g., numbers potentially eligible, examined for eligibility, confirmed eligible, included in the study, completing follow-up, and analysed.	Partially – Final analyzed sample described, but exact number of initially screened patients is not provided.
Descriptive data	14	Give characteristics of study participants and information on exposures and potential confounders.	Yes – Tables include detailed patient and treatment characteristics.
Outcome data	15	Report numbers of outcome events or summary measures over time.	Yes – Outcome data are presented as VAS scores and statistical comparisons.
Main results	16	Give unadjusted estimates and, if applicable, confounder-adjusted estimates and their precision (e.g., 95% confidence interval).	Yes – Confidence intervals and significance levels are provided for all comparisons.
Other analyses	17	Report other analyses done—e.g., subgroup analyses and sensitivity analyses.	Yes – Comparison with previous profile-only data and subgroup analyses by rater type are provided.
**Discussion**			
Key results	18	Summarise key results with reference to study objectives.	Yes – Results are summarized clearly in the discussion and conclusions.
Limitations	19	Discuss limitations of the study, taking into	Yes – Limitations such as lack of a

		account sources of potential bias or imprecision.	priori power calculation and generalizability are well discussed.
Interpretation	20	Give a cautious overall interpretation of results considering objectives, limitations, and results from similar studies.	Yes – Interpretation is cautious, contextualized with prior studies.
Generalisability	21	Discuss the generalisability (external validity) of the study results.	Yes – The authors discuss applicability within white European populations and note the need for further studies.
**Other information**			
Funding	22	Give the source of funding and the role of the funders for the present study.	Yes – The study reports no external funding.
